# Functional Up-Conversion Nanoparticle-Based Immunochromatography Assay for Simultaneous and Sensitive Detection of Residues of Four Tetracycline Antibiotics in Milk

**DOI:** 10.3389/fchem.2020.00759

**Published:** 2020-10-08

**Authors:** Ying Xu, Biao Ma, Erjing Chen, Xiaoping Yu, Chuanxin Sun, Mingzhou Zhang

**Affiliations:** ^1^Zhejiang Provincial Key Laboratory of Biometrology and Inspection & Quarantine, China Jiliang University, Hangzhou, China; ^2^Department of Plant Biology, Uppsala BioCenter, Linnean Center for Plant Biology, Swedish University of Agricultural Science (SLU), Uppsala, Sweden

**Keywords:** up-conversion nanoparticle, tetracycline, immunoassay, multi-residue detection, milk

## Abstract

An ultrahigh-sensitivity lateral flow immunochromatography (LFIC) assay based on up-converting nanoparticles (UCNPs) was developed to carry out a multi-residue detection of tetracycline in milk. The sensitivity of the immunoassay was greatly improved by the use of a broad-spectrum monoclonal antibody attached to UCNPs to form a signal probe. Under the optimal conditions, the UCNP-LFIC assay enabled sensitive detection of tetracycline (TC) as well as of oxytetracycline (OTC), chlortetracycline (CTC), and doxycycline (DOX) within 10 min, with *IC*_50_ values of 0.32, 0.32, 0.26, 0.22 ng/mL, respectively. There was no cross-reactivity with ten other antibiotics. Similarly, we evaluated the experimental results for matrix effects. Experiments involving spiking showed the four tetracycline antibiotics displaying mean recoveries ranging from 93.95 to 111.90% with relative standard deviations (RSDs) of < 9.95%. The detection results of actual samples using the developed method showed a good correlation (*R*^2^ ≥ 0.98) with the results using high-performance liquid chromatography (HPLC). Thus, the assay can achieve an ultrahighly sensitive detection of antibiotics in milk, and can hence promote human health and provides promising applications in the bio-detection field.

## Introduction

As antibacterial pharmaceuticals, antibiotics have been widely used to treat bacterial infections in humans and animals (Han et al., 2015; Dubald et al., 2018). For example, β-lactams, tetracycline (TC) and streptomycin are the most commonly used antibiotics for the treatment of cow mastitis (Zhou et al., 2018). As a broad-spectrum antibiotic, tetracycline has antibacterial effects and shows widespread use in milk (Xie et al., 2018). But overuse of this antibiotic may cause gastrointestinal tract problems such as nausea and vomiting and can also be toxic to the liver and cause kidney damage, allergic reactions, etc. (Wang P. L. et al., 2015; Yang et al., 2017). In this regard, the United States, the European Union and other countries have stipulated the maximum residue limits (MRLs) of tetracyclines compounds in milk as well as in animal tissues used as food to be 0.1 mg/L (European Union, 2010; Food and Drug Administration U.S. Department of Health and Human Services, 2018). China also clearly stipulates MRLs of 100 μg/kg for tetracyclines in milk (GB, 2019).

To date, multiple analytical methods have been used for the analysis of antibiotics, with these methods including high-performance liquid chromatography (HPLC) (Deng et al., 2016), ultra-performance liquid chromatography-tandem mass spectrometry (UPLC-MS/MS) (Tahrani et al., 2018; Wang et al., 2018) and others. Although the above-mentioned methods have the advantages of high accuracy and sensitivity, they also still display some disadvantages such as complex operations, high analytical cost and time-consuming sample pretreatment, which limit their applications for on-site analysis. Therefore, various immunoassays, including enzyme-linked immunosorbent assay (Wei et al., 2018; Liu et al., 2019), lateral-flow immunochromatography (LFIC) (Li et al., 2010), and other novel immune sensors (Fernández et al., 2010; Zeng et al., 2019) have been developed for the detection of various antibiotic residues in milk. Of these assays, LFIC is the simplest method and the most rapid to carry out (Vanrell et al., 2013). Most LFIC experiments employ colloidal gold (CG) as labels to achieve qualitative or quantitative analysis of analytes (CháferPericás et al., 2010). However, CG-LFIC still displays some problems; for example, the detection results are easily interfered with by colored samples, and the detection sensitivity is low (Byzova et al., 2014; Zhu et al., 2014).

In recent years, the emergence of fluorescent materials has greatly improved the sensitivity of detection of antibiotic residues (Gu and Zhang, 2018). Here, up-conversion nanomaterials constitute one of the most popular types of fluorescent materials. Up-conversion nanoparticles (UCNPs) are novel lanthanide-doped fluorescence materials, and they can convert a near-infrared light excitation to an emission of visible light. UCNP-based probes displaying many charming features, such as high photochemical stability, narrow emission, low toxicity, low background fluorescence, and a multicolor tunable spectrum, have been developed (Li et al., 2019; Ummi and Siddiquee, 2019; Zhang et al., 2019; Liu et al., 2020). Compared with CG, UCNPs have shown higher sensitivity and accuracy levels due to the optical properties of their inorganic inert materials (Lan et al., 2017). Therefore, UCNP-LFIC assays have become bioassay research focuses (Dai et al., 2016; You et al., 2017; Zhou et al., 2018; Perry et al., 2019). UCNP-LFIC analysis has been reported to be applied relatively effectively in the detection of small molecules such as aflatoxin B1 (Zhao et al., 2016) and clenbuterol (Wang S. et al., 2015) as well as organophosphorus pesticides (Tao et al., 2019). However, no reports of its use in antibiotic testing have been published to the best of our knowledge.

In this study, a novel UCNP-LFIC analysis has been developed for a rapid, highly sensitive quantitative detection of TC in milk. At the same time, the tolerance of this analysis to a variety of dairy product matrix interferences was tested, and our results showed the ability of this analysis to screen out TC within 10 min. Therefore, the UCNP-LFIC analysis is expected to have an important role in food safety testing applications.

## Materials and Methods

### Reagents and Chemicals

Tetracycline (TC), chlortetracycline (CTC), oxytetracycline (OTC), doxycycline (DOX), kanamycin (KAN), streptomycin (SM), enrofloxacin (ENR), penicillin (PEN), florfenicol (FFC), thiamphenicol (TAP), gentamicin (GEN), erythromycin (ERY), sulfamethazine (SMZ), and lincomycin (LIN) standards were purchased from the National Institute of Metrology, P. R. China (Beijing, China). Carboxylic acid-functionalized UCNPs (NaYF_4_: Yb3^+^, Er3^+^), with an average diameter of 30 nm and displaying an excitation spectrum peak at a wavelength of 980 nm and emission spectrum peak at 475 nm, were obtained from Fluo Nanotech Co., Ltd (Hangzhou, China). 1-(3-Dimethylaminopropyl)-3-ethylcarbodiimide hydrochloride (EDC) and N-hydroxysuccinimide (NHS) were purchased from Sigma-Aldrich (St. Louis, MO, USA). Human serum albumin (HSA) and bovine serum albumin (BSA) were obtained from Sino-American Biotechnology (Luoyang, China). Goat anti-mouse IgG, Tween-20, and Freund's complete and incomplete adjuvants (cFA and iFA) were obtained from Aladdin Industrial Corporation (Shanghai, China). 2-(N-morpholino) ethanesulfonic acid monohydrate (MES) was supplied by Sangon Biotech Co., Ltd (Shanghai, China). Cells of the myeloma cell line of Sp2/0 were obtained from the Chinese Academy of Sciences (Shanghai, China). Nitrocellulose membrane, glass fiber used as a sample pad and conjugate pad, and cotton pulp used as an absorbent pad were all obtained from Millipore Corp (USA). All other reagents were of analytical grade and obtained from Sinopharm Group Chemical Reagent Co., Ltd. (Shanghai, China).

Phosphate buffer solution (PBS, 0.01 M, pH 7.4) was prepared by weighing 0.2 g of NaCl, 1.55 g of NaH_2_PO_4_ and 0.25 g of KH_2_PO_4_ in ultrapure water to a final volume of 1 L, and then adjusting the pH to 7.4 with NaOH. Borate buffer solution (BBS, 0.05 M, pH 8.2) was prepared by weighing 0.81 g of boric acid and 0.67 g of NaB_4_O_7_•10H_2_O in ultrapure water to a final volume of 1 L containing 0.05% (v/v) Tween-20. Carbonate buffer solution (CBS, 0.1 M, pH 9.6) was prepared by weighing 1.59 g of Na_2_CO_3_ and 2.94 g of NaHCO_3_ in ultrapure water to a final volume of 1 L. MES buffer (0.05 M, pH 6.5) was prepared by weighing 9.76 g of MES in ultrapure water to a final volume of 1 L containing 0.05% (v/v) Tween-20, and then adjusting the pH to 6.5 with NaOH.

### Apparatus

An XYZ3000 dispensing platform and CM2000 guillotine cutter (BioDot, Irvine, CA, USA) were used to prepare the immunochromatographic strip. A Hitachi F-4500 fluorescence spectrometer system (Hitachi, Tokyo, Japan) was used to record the fluorescence spectrum. The UCNP-based LFIC (UCNP-LFIC) strips were scanned using a strip reader (Suzhou Helmen Precise Instruments, Suzhou, Jiangsu, China) with 980-nm-wavelength near-infrared laser excitation.

### Preparation of TC-BSA and TC-HSA

The complete antigens were prepared by consulting relevant literature (Liu et al., 2012; Chen et al., 2019). The TC-HSA and TC-BSA were used as the immunogen and coating antigen, respectively. Briefly, to prepare TC-BSA, a mass of 2 mg of TC was dissolved in 200 μL of DMF (0.01 M), and then to this solution were added 4 mg of NHS and 2.5 mg of EDC. The resulting mixture was stirred at 25°C overnight, and a mass of 40 mg of BSA (in 2 mL 0.1 M pH 9.6 CBS) was added to the stirred mixture. The mixture was reacted at 25°C for 4 h in a light-proof set up. The final conjugates were dialyzed at 4°C for 72 h against the PBS buffer (0.01 M, pH 7.4). The collected samples were stored at −20°C until use. The method used to prepare the TC-HSA conjugate was essentially the same as that used to prepare TC-BSA, except of course using HSA instead of BSA. In addition, the coupling products were identified from the results of SDS-PAGE electrophoresis and UV-Vis spectroscopy to ensure the success of the coupling process.

### Production of Monoclonal Antibody

Antibodies were produced by immunizing BALB/c mice according to the previous work in our laboratory (Gong et al., 2014). Briefly, TC-HSA was emulsified with an equal amount of adjuvant (first with Freund's complete adjuvant and secondarily with Freund's incomplete adjuvant) and then injected subcutaneously into the mice. After three immunizations, serum titers were determined by performing enzyme-linked immunosorbent assay investigations. When the serum titer no longer increased, the spleen cells of the immunized mice were removed and fused with the Sp2/0 myeloma cells. The resulting hybridoma cells, which secreted TC monoclonal antibody, were screened by using HAT and HT medium. Then the monoclonal cell strain was injected intraperitoneally into mice to produce ascites. Finally, the ascites was purified to obtain monoclonal antibodies against TC.

### Preparation of Colloidal Gold-mAb Probes

Based on the procedures used in our previous work (Gong et al., 2014), colloidal gold (CG) nanoparticles were prepared with an average diameter of 20 nm and coupled with above-described monoclonal antibody against TC.

### Preparation of UCNP-mAb Probes

A modified version of previously used protocols (Yeo et al., 2017) was used here to prepare the UCNP-mAb probes. Briefly, a mass of 1 mg of carboxylic UCNPs was dissolved in 400 μL of a MES (0.05 M, pH 6.5) solution, and then activated by adding 15 μL of EDC (10 mg/mL) to the resulting solution. After 30 min of incubation with slow shaking at room temperature (RT), the activated product was centrifuged at 13,000 rpm for 30 min to remove EDC, and dissolved in 500 μL of BBS (0.05 mol/L, pH 8.2, containing 0.05% Tween-20). To five samples of this solution were added volumes of 1 ml of anti-TC mAb at concentrations of 2.5, 5, 10, 20, and 40 μg/mL, respectively, and the resulting mixtures were stirred softly at RT for 2 h. At the end of the reaction, a volume of 55 μL of 10% BSA (w/v) as a blocking buffer was added to each stirred mixture. Then, after 2 h, each resulting complex was centrifuged two times at 13,000 rpm to remove unreacted antibodies and BSA. Finally, the precipitates were resuspended in 500 μL of the preservation solution (0.05 M, pH 8.2) for storage at 4°C until further use.

### Preparation of Lateral Flow Strips

Each lateral flow strip consisted of five parts: a sample pad, conjugate pad, nitrocellulose membrane, absorbent pad and backing card. The sample and conjugate pads were immersed in PBS buffer (0.05 M, pH 7.4, containing 1% (w/v) BSA, and 0.05% (v/v) Tween-20) for 30 min, and then placed in a desiccator at 37°C overnight and afterwards stored in a sealed bag before use. The conjugate pad was lyophilized for 2 h and sprayed with labeled mAb conjugate that was diluted with BBS buffer (0.05 M, pH 8.2, containing 8% (w/v) sucrose, and 1% (w/v) BSA). TC-BSA and goat anti-mouse IgG were painted on the nitrocellulose membranes as, respectively, test and control lines separated from each other by 3 mm. The membrane was treated with PBS buffer (0.01 M, pH 7.4, containing 1% (w/v) BSA), and dried for 12 h at 37°C and stored in a dry bag. The absorbent pad was stored at RT without any treatment. Finally, all of the above components were assembled on a backing card in a certain order, cut into 2.5 mm strips and kept at RT.

### Optimization of the UCNP-LFIC Test Strip

Various factors were optimized based on their effects on fluorescence intensity, *IC*_50_ value (the ratio of the Test/Control (T/C) value of the 50% inhibition concentration to the T/C value of the blank), and sensitivity. The factors that were optimized included the immunoreaction time, the concentration of labeled antibody, identity of the buffer, and the concentrations of coating antigen and goat anti- mouse-HRP.

### Detection Procedure Using the UCNP-LFIC Strip

Specific amounts of UCNP-mAb probe and BBS buffer were uniformly mixed and dropped onto the sample pad. The result of the reaction after a specified period of time was observed by using a diode generating a 980-nm-wavelength laser. At the same time, the above-described lateral flow strips were placed into the up-conversion luminescence reader, followed by recording the fluorescence intensities of the test and control lines.

### Assessment of Specificity and Sensitivity

Standard curves were established by analyzing a series of concentrations (0, 0.1, 0.25, 0.5, 1, 2.5, 5, 10, and 20 ng/mL) of each of the four TCs and their mixed solutions one by one. The *IC*_50_ was used to evaluate the sensitivity of the assay.

To assess the specificity of the developed strip, fourteen different antibiotics were tested, including TC, CTC, OTC, DOX, KAN, SM, ENR, PEN, FFC, TAP, GEN, ERY, SMZ, and LIN. Each standard antibiotic solution was diluted to a final concentration of 500 ng/mL as described above and tested separately.

### Experiments for Spiked Recovery and Actual Sample Analysis

For experiments involving spiking, spiked samples were prepared by adding different concentrations of analytes (10, 20, 40, 80 ng/mL) to blank milk samples, and analyzed by using the developed UCNP-LFIC strip.

For actual sample analysis, 36 milk samples collected from a local market were analyzed by using both the UCNP-LFIC strip and HPLC at the same time, and the results of these analyses were compared.

### Statistical Analysis

The peak value of each fluorescence spectrum was analyzed by using Origin 9 software (Origin Lab., USA); The standard curves were analyzed and the best reaction time, coating concentration, and buffer were determined using Microsoft Excel software (Microsoft Inc., USA). The chart of the test strip was drawn with Photoshop software (Adobe Systems, USA).

## Results and Discussion

### Detection Principle

**Figure 1:**
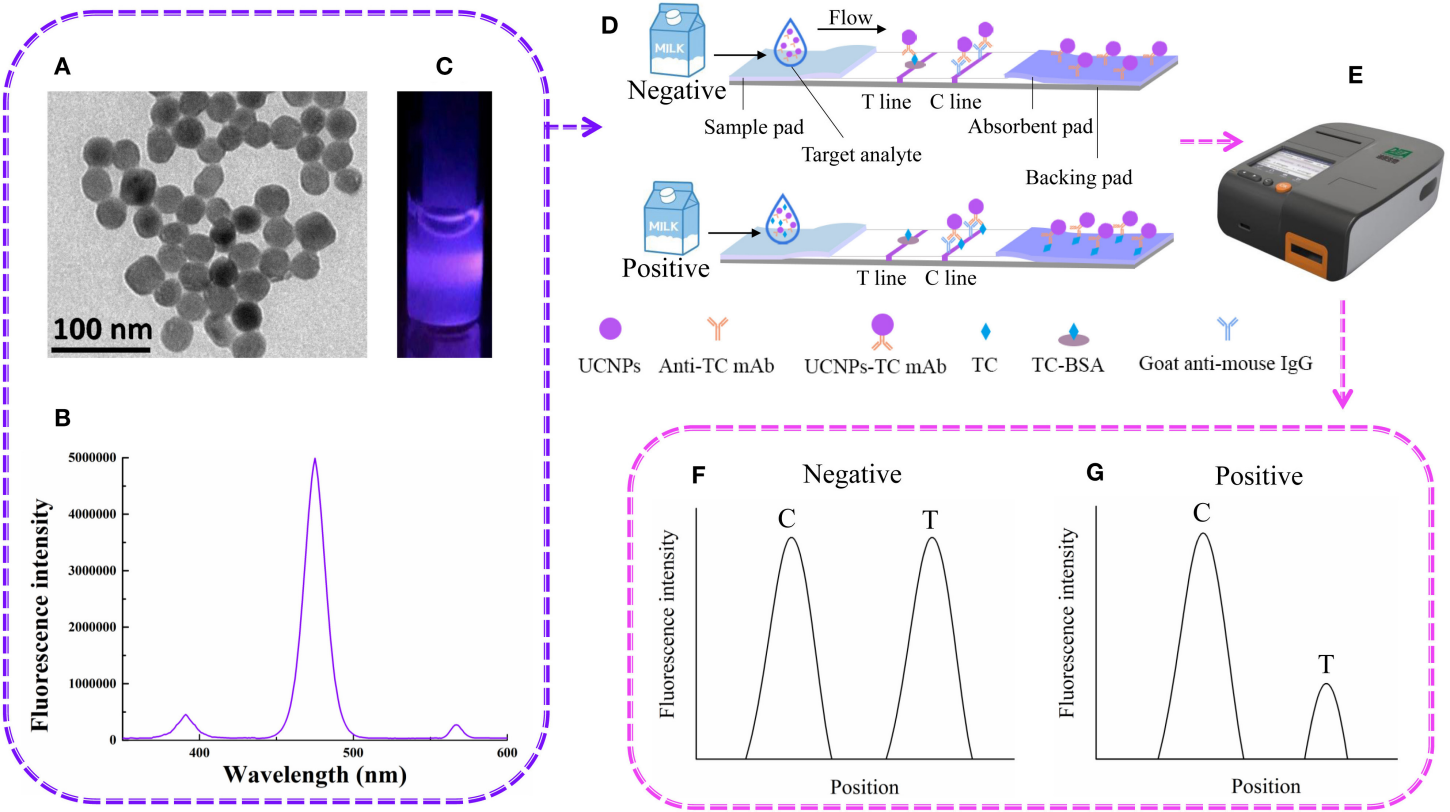
The UCNP-LFIC detection principle. **(A)** TEM images of UCNPs. **(B)** Emission spectra of carboxylic-acid-functionalized UCNPs. **(C)** Appearance of the UCNPs under irradiation with 980-nm-wavelength light. **(D)** Schematic diagram showing how UCNP-LFIC functions. **(E)** The UCNP-LFIC reader (excitation/emission wavelengths of 980/804 nm). **(F,G)** Test results of the UCNP-LFIC reader test results.

The detection of UCNP-LFIC was based on the principle of a competitive reaction. As shown in Figure 1, the UCNP-mAb probe was uniformly mixed with the solution containing the target analyte. (TEM image, emission spectrum of carboxylic acid-functionalized UCNPs and the appearance of the strip under 475-nm-wavelength radiation are shown in Figures 1A–C, respectively). When the mixed solution was added to the sample pad, the solution quickly flowed through the strip as a result of capillary forces (Figure 1D). After the reaction, the signal intensity was read with a portable reader (Figure 1E). The immobilized antigen competed with the target analyte in the sample solution for the UCNP-mAb probe. When the target analytes were combined with the probe, a UCNP-mAb-analyte complex was formed, and the amounts of UCNP-mAb probes captured by the test line decreased, indicating a positive result, as shown in Figure 1G. If the target analytes were absent or not below the detection range in the sample solution, all UCNP-mAb probes would react with the test line. The excess UCNP-mAb probes would continue to flow through the membrane and control line. A negative result is shown in Figure 1F.

### Characterization of Antigens and Antibodies

Tetracycline is a low-molecular-weight antibiotic and is not immunogenic, so it must be coupled to a carrier protein to be immunogenic. In this study, we performed N-hydroxysuccinate active esterification to connect TC hapten with carrier proteins BSA and HSA to prepare artificial antigens.

In the SDS-PAGE identification results for antigens and antibodies, the TC-BSA band lagged behind the BSA band. This result indicated that the TC successfully coupled to BSA. The characteristic TC-HSA band also indicated that TC successfully coupled to HSA. These results are shown in Supplementary Figure 1. The results of UV-Vis spectroscopy investigations of the antigens are shown in Supplementary Figure 2. The characteristic TC peaks were observed at wavelengths of 274 and 350 nm. A characteristic BSA peak was found at 275 nm. However, the characteristic TC-BSA speaks were observed at 278 and 358 nm. These results suggested that TC successfully coupled to the BSA carrier protein. Similarly, TC-HSA was also successfully synthesized.

Several stable cell lines were selected by performing cell fusion, and antibodies were obtained after purification of ascites. The indirect competition enzyme-linked immunosorbent assay (ELISA) was used to assess the activities of these antibodies and to pick out the most active antibody. Cross-reactivity experiments of this antibody with ten other different antibiotics (KAN, SM, ENR, PEN, FFC, TAP, GEN, ERY, SMZ, and LIN) showed good specificity (Supplementary Table 1).

### Optimization of UCNP-LFIC Test Strip Parameters

For a rapid and sensitive immunoassay, the selection and optimization of an appropriate detection system is crucial. The effects of certain parameters, in particular the immunoreaction time, concentration of labeled antibody, identity of the buffers, and concentrations of TC-BSA, and goat anti- mouse-HRP, on the performance of the system were examined.

**Figure 2:**
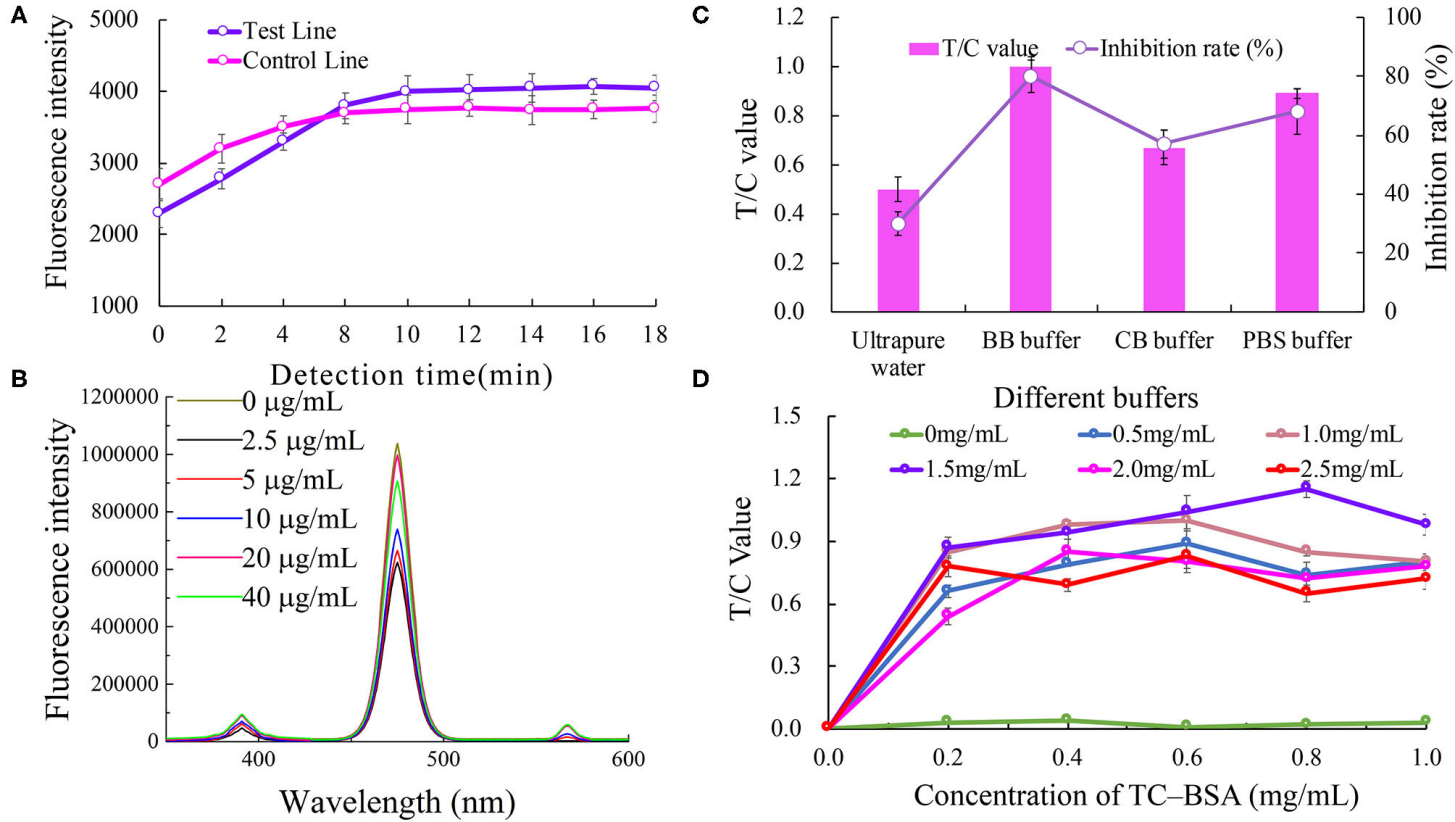
Optimization of the UCNP-LFIC assay. **(A)** Effect of immune response time on the biosensor fluorescence intensity. **(B)** Effect of different antibody concentrations on the fluorescence intensity of mixed solutions. **(C)** Effect of type of buffers. **(D)** Effect of TC-BSA and goat anti- mouse-HRP concentrations.

The effect of the immune response time on the biosensor fluorescence intensity is shown in Figure 2A. The fluorescence intensities of the C line and T line gradually increased with the increasing immune reaction time for the first 10 min, and tended to be gentle after 10 min. Therefore, a reaction time of 10 min was considered to be the best immune response time.

The effect of anti-TC mAb concentration on the fluorescence intensity of the mixed solutions is shown in Figure 2B. No direct connection between the two was found. Nevertheless, when the concentration of the antibody was 20 μg/mL, the fluorescence intensity was the highest. Therefore, the optimal concentration of anti-TC mAb was considered to be 20 μg/mL.

The analytical performance of the LFIC was greatly affected by the choice of buffer. We tested four different buffers, namely ultra-pure water (pH 5.0), BBS buffer (0.05 M, pH 8.2), PBS buffer (0.05 M, pH 7.4), and CBS buffer (0.05 M, pH 9.6). The results are shown in Figure 2C. By comparing the T/C values and inhibition rates, the BBS buffer was concluded to be the best of the tested buffers.

The concentrations of TC-BSA and goat anti- mouse-HRP coated on the NC membrane were optimized at the same time. The concentrations of the coated antigen tested were 0, 0.2, 0.4, 0.6, 0.8, and 1.0 mg/mL, respectively, and the concentrations of goat anti- mouse-HRP tested were 0, 0.5, 1, 1.5, 2.0, and 2.5 mg/mL, respectively. According to the results (Figure 2D), and considering the value of T/C, 0.8 mg/mL of TC-BSA and 1.5 mg/mL of goat anti-mouse-HRP were used as the optimum amounts in this study.

We found the detection time of this study was much shorter than those reported elsewhere from the optimization results of inspection time (Zou et al., 2019). This advantage has laid a good foundation for future research. In addition, the concentrations of TC-BSA and goat anti-mouse-HRP coated on the NC film optimized in this experiment were higher than those reported elsewhere (Zhao et al., 2016), which may be an important contributor to the higher fluorescence intensity value of the entire current optimized system. To take costs into consideration and yet achieve best results, we plan to reduce the amount of TC-BSA and goat anti-mouse-HRP coated on the NC membrane in subsequent research.

### Sensitivity and Specificity Analyses of UCNP-LFIC

CG-LFIC and UCNP-LFIC assays were established based on the competitive form after system optimization. The CG-LFIC assay was optimized according to methods previously developed in our laboratory (Dai et al., 2015).

**Figure 3:**
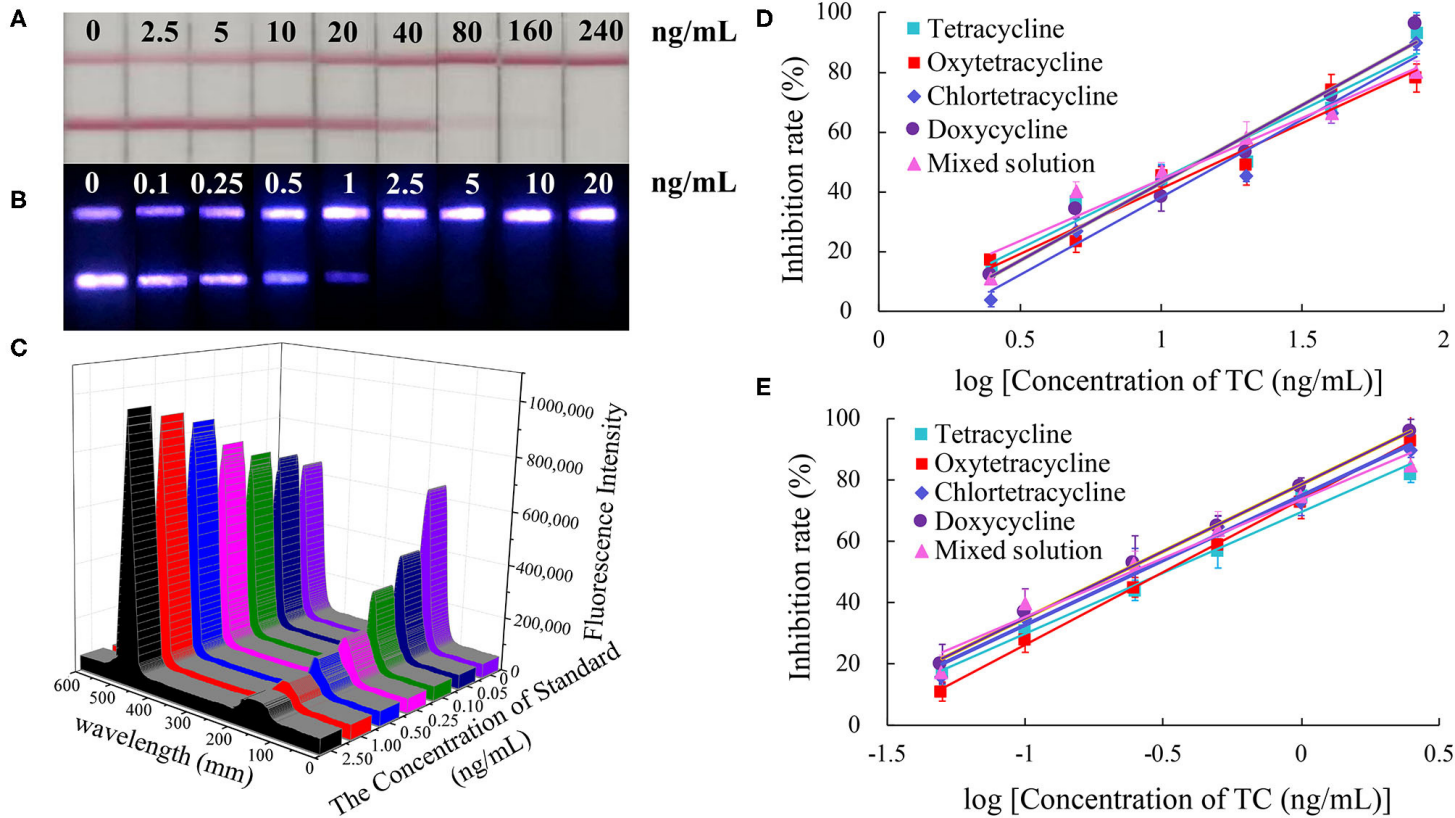
Comparison of the sensitivity analysis between CG-LFIC and UCNP-LFIC. **(A)** Sensitivity analysis of TC by CG-LFIC. **(B)** Sensitivity analysis of TC by UCNP-LFIC. **(C)** Emission spectra of sensitivity analysis by UCNP-LFIC. **(D,E)** The standard curve for TC by CG-LFIC and UCNP-LFIC.

The color of the test line disappeared at a concentration of 80 ng/mL of standard solution in the CG-LFIC analysis. Under the same conditions, the test line when performing the UCNP-LFIC analysis almost disappeared at 2.5 ng/mL (Figures 3A,B). Figure 3C showed the emission spectra of sensitivity analysis by UCNP-LFIC. Based on the data acquired using the UCNP-based LFIC strip reader, the corresponding standard curves of CG-LFIC and UCNP-LFIC were established (Table 1 and Figures 3D,E). The results showed that in this study, UCNPs were more suitable as label than was traditional CG. This result may have been due to the characteristics of the material itself.

**Table 1:** Linear regression equations based on CG-LFIC and UCNP-LFIC analyses of the TC antibiotic.

**CG-LFIC**			
**Antibiotic**	**Standard curve**	***R**^**2**^*	**IC**_**50**_ **(ng/mL)**
Tetracycline	*y =* 46.41x – 2.23	0.95	13.34
Oxytetracycline	*y =* 43.85x – 2.78	0.96	15.98
Chlortetracycline	*y =* 52.01x – 13.84	0.96	16.88
Doxycycline	*y =* 52.11x – 9.11	0.96	13.63
Mixed solution	*y =* 41.19x + 2.94	0.94	13.88
**UCNP-LFIC**			
**Antibiotic**	**Standard Curve**	***R**^**2**^*	**IC**_**50**_ **(ng/mL)**
Tetracycline	*y =* 39.65x + 69.55	0.98	0.32
Oxytetracycline	*y =* 47.38x + 73.66	0.99	0.32
Chlortetracycline	*y =* 42.14x + 74.87	0.99	0.26
Doxycycline	*y =* 43.65x + 78.58	0.99	0.22
Mixed solution	*y =* 38.32x + 73.67	0.97	0.24

As a new type of fluorescent material, the up-conversion nanomaterial was found to display unique optical properties, unmatched by traditional CG. Therefore, the UCNP can be used as an alternative to colloidal gold, and greatly improve the detection sensitivity (Shen et al., 2014).

**Figure 4:**
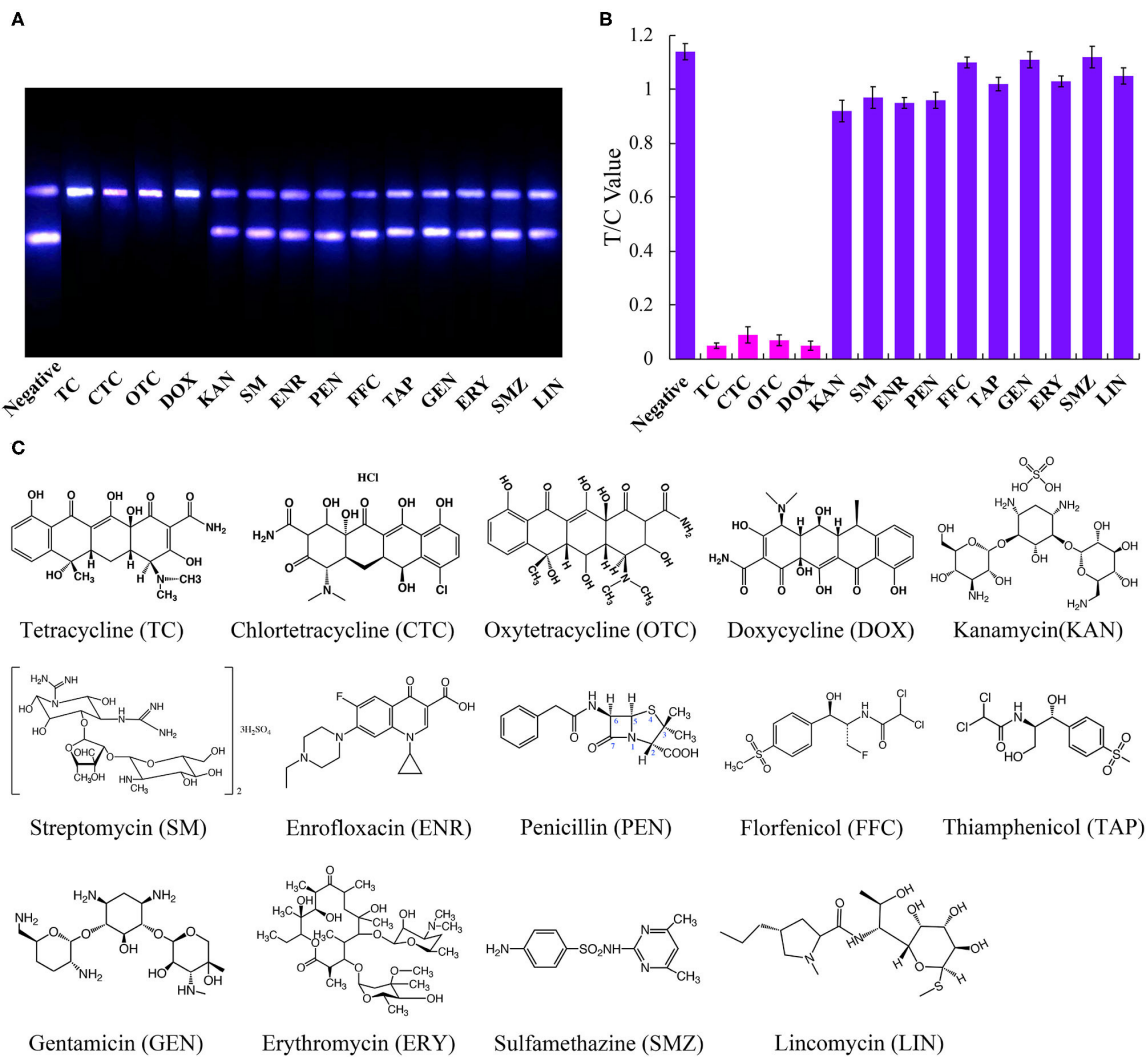
Specific analysis of UCNP-LFIC. **(A)** Specific evaluation results (from left to right are negative, TC, CTC, OTC, DOX, KAN, SM, ENR, PEN, FFC, TAP, GEN, ERY, SMZ, and LIN). **(B)** T/C value data analysis showing the extents of cross-reactivity of TC with other antibiotics. **(C)** Molecular structures of the antibiotics.

To determine the specificity of the immunoassay, the above-mentioned fourteen antibiotics were each diluted from a standard solution to a final concentration of 500 ng/mL and using the UCNP-LFIC assay. The rates of inhibition afforded by TC, CTC, OTC, and DOX were significantly higher than those from the other ten antibiotics (Figure 4). These results showed the selectivity of the assay to be consistent with the mAb specificity reported in the literature (Han et al., 2015; Yan et al., 2015; Tan et al., 2016; Wang et al., 2016).

### Matrix Interference in Real Samples

Complex components in a sample, such as proteins, saccharides and polyphenols, can interfere with the detection of the target analytes. Carrying out continuous dilution to reduce these interferences is in general a very simple and effective method for overcoming this issue.

**Figure 5:**
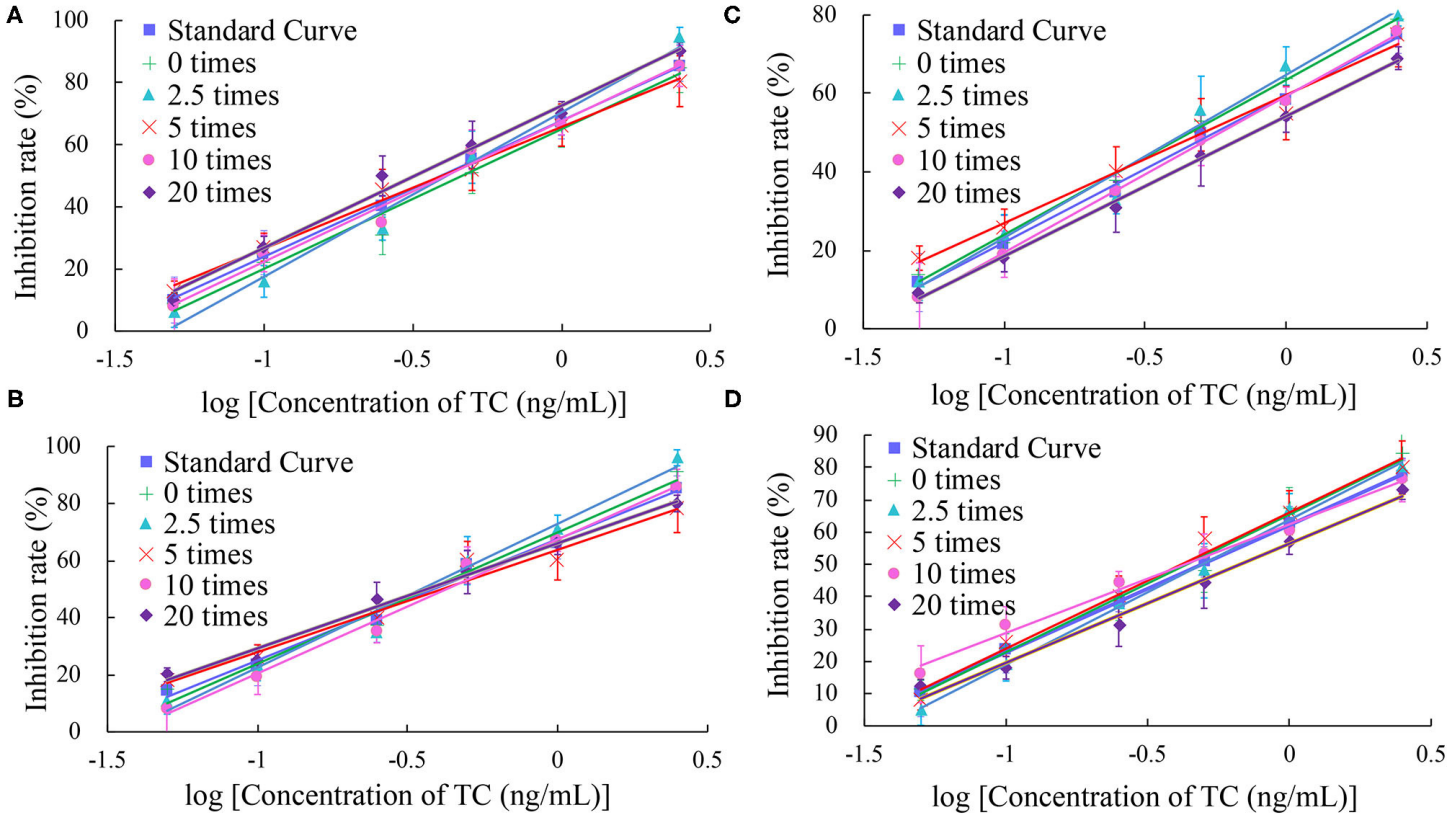
Fitted standard curve results of TC at different dilution times in real matrix samples matrix. **(A)** Fresh milk. **(B)** Pure milk. **(C)** Sweet milk. **(D)** Reconstituted milk.

In this study, the impact of the matrix in four milk products (fresh, pure, sweet, and reconstituted milk) on the sensitivity of the UCNP-LFIC assay was evaluated. Matrix effects were eliminated and the accuracy of the method was ensured by serially diluting the sample before carrying out the detection experiment. In short, the four milk samples were each diluted by 0-, 2.5-, 5-, 10-, and 20-fold with BBS buffer (0.05 M, pH 8.2) and made into standard solutions. At 5-fold dilution, the matrix effect values of the four milk samples were between 84.59 and 103.52% (Table 2 and Figure 5) and the matrix dilution could be ignored (Matuszewski et al., 2003).

**Table 2:** Matrix effect of different milk products.

**Sample**	**Dilution**	**Standard curve**	***R^**2**^***	**IC_**50**_ (ng/mL)**	**Matrix effect**
Fresh milk	1	*y =* 44.97*x* + 65.03	0.98	0.46	120.89%
	2.5	*y =* 52.93*x* + 70.42	0.98	0.41	107.57%
	5	*y =* 39.17*x* + 65.74	0.99	0.39	103.52%
	10	*y =* 45.30*x* + 67.61	0.98	0.40	106.52%
	20	*y =* 45.80*x* + 72.58	0.98	0.32	83.86%
Pure milk	1	*y =* 45.99*x +* 69.84	0.98	0.37	86.90%
	2.5	*y =* 50.31*x +* 72.86	0.98	0.36	85.21%
	5	*y =* 35.83*x +* 63.76	0.97	0.41	96.94%
	10	*y =* 46.90*x* + 67.36	0.98	0.43	100.01%
	20	*y =* 36.80*x* + 66.05	0.99	0.36	85.21%
Sweet milk	1	*y =* 39.50*x* + 63.47	0.99	0.46	81.64%
	2.5	*y =* 41.53*x* + 64.77	0.98	0.44	79.14%
	5	*y =* 32.69*x* + 59.62	0.98	0.51	90.92%
	10	*y =* 39.88*x* + 59.32	0.99	0.58	104.54%
	20	*y =* 35.68*x* + 54.19	0.99	0.77	137.63%
Reconstituted milk	1	*y =* 42.61*x* + 65.43	0.99	0.43	88.32%
	2.5	*y =* 44.79*x* + 63.78	0.99	0.49	100.08%
	5	*y =* 42.13*x* + 66.04	0.98	0.42	84.59%
	10	*y =* 33.61*x* + 62.39	0.99	0.43	87.98%
	20	*y =* 36.87*x* + 56.41	0.98	0.67	136.38%

### Detection of Spiked Analytes

Experiments involving spiking milk with TC, CTC, OTC, or DOX analytes were performed to evaluate the accuracy and repeatability of UCNP-LFIC assay. To respective blank milk samples were added different concentrations of an analytes, and six replicates of each concentration were tested. Relative standard deviations (RSDs) were calculated to assess the accuracy of the method and the repeatability between batches. The results are shown in Table 3. For the intra-assay, the recovery rates ranged from 86.85 to 112.08% with RSDs ≤ 7.88%. For the inter-assay, the recovery rates ranged from 93.95 to 111.90% with RSDs ≤ 9.95%, values satisfying the general requirements of trace detection. The above results indicated the UCNP-LFIC assay to be suitable for quantitative analysis of milk.

**Table 3:** Recovery rate determination of TC in milk samples from UCNP-LFIC analysis.

**Analyte**	**Spiked level (ng/mL)**	**Intra-assay^a^**			**Inter-assay^a^**		
		**Detected amount (ng/mL)**	**Recovery rate**	**RSD (*n =* 6)**	**Detected amount (ng/mL)**	**Recovery rate**	**RSD (*n =* 6)**
TC	10	10.47	104.70%	2.48%	11.19	111.90%	7.19%
	20	18.59	92.95%	3.86%	19.87	99.35%	5.46%
	40	44.32	110.80%	6.03%	41.98	104.95%	6.34%
	80	88.38	110.48%	4.13%	83.76	104.70%	6.39%
CTC	10	11.03	110.30%	3.31%	10.82	108.20%	6.35%
	20	20.49	102.45%	3.51%	18.86	94.30%	5.86%
	40	37.81	94.53%	2.30%	37.67	94.18%	4.92%
	80	76.96	96.20%	3.55%	79.43	99.29%	6.70%
OTC	10	9.87	98.70%	3.48%	11.07	110.70%	9.10%
	20	21.38	106.90%	6.37%	18.79	93.95%	7.11%
	40	40.60	101.50%	7.88%	38.88	97.20%	9.95%
	80	77.56	96.95%	5.81%	80.74	100.93%	6.30%
DOX	10	9.16	91.60%	7.83%	10.45	104.50%	3.31%
	20	17.37	86.85%	4.49%	19.08	95.40%	7.34%
	40	44.83	112.08%	4.76%	43.25	108.13%	2.93%
	80	81.63	102.04%	1.64%	83.74	104.68%	2.73%

a *Repeat assay (n = 6)*.

Although UCNP-LFIC analysis cannot distinguish between tetracycline, oxytetracycline, chlortetracycline and doxycycline, it can be used as a screening tool for the residues of the four tetracyclines antibiotics in any case, which is expected to help in future research.

### Detection of Actual Samples

To further validate the developed UCNP-LFIC assay, 36 milk samples collected from all around the world were analyzed using both the UCNP-LFIC assay and HPLC method (Table 4). Nineteen samples were determined according to the UCNP-LFIC assay to be negative for the tetracycline antibiotics, and the same nineteen samples were also indicated to be negative from the HPLC results. For the sake of the accuracy of the results, each sample was analyzed at least three times. The testing time and procedures of the UCNP-LFIC were shorter and more convenient than those of other methods (Iwaki et al., 1992), and the results were obtained by a visual assessment that could be reliably applied to the detection of antibiotics in real samples.

**Table 4:** Detection of TC in milk samples from UCNP-LFIC with HPLC.

**Sample**	**UCNP-LFIC^a^ (ng/mL)**	**HPLC^a^ (ng/mL)**	**Sample**	**UCNP-LFIC^a^ (ng/mL)**	**HPLC^a^ (ng/mL)**
Milk 01	11.69 ± 0.27	13.44 ± 0.48	Milk 19	18.39 ± 0.69	19.12 ± 0.81
Milk 02	13.46 ± 0.14	15.72 ± 0.35	Milk 20	/	/
Milk 03	/^b^	/	Milk 21	/	/
Milk 04	20.23 ± 1.06	19.84 ± 0.39	Milk 22	/	/
Milk 05	/	/	Milk 23	23.24 ± 1.27	22.98 ± 0.39
Milk 06	/	/	Milk 24	11.42 ± 0.89	12.37 ± 1.02
Milk 07	14.29 ± 0.36	16.37 ± 0.71	Milk 25	/	/
Milk 08	18.94 ± 2.01	18.36 ± 1.47	Milk 26	/	/
Milk 09	/	/	Milk 27	/	/
Milk 10	14.83 ± 0.38	15.17 ± 0.08	Milk 28	/	/
Milk 11	24.58 ± 1.09	25.26 ± 1.27	Milk 29	16.71 ± 0.33	15.49 ± 0.62
Milk 12	30.88 ± 2.27	33.69 ± 0.41	Milk 30	13.34 ± 1.42	14.82 ± 0.73
Milk 13	10.93 ± 1.01	11.62 ± 0.58	Milk 31	/	/
Milk 14	/	/	Milk 32	/	/
Milk 15	40.98 ± 3.32	39.09 ± 0.75	Milk 33	15.67 ± 1.24	17.47 ± 1.15
Milk 16	/	/	Milk 34	/	/
Milk 17	/	/	Milk 35	/	/
Milk 18	16.64 ± 2.15	17.93 ± 1.03	Milk 36	/	/

a *Repeat assay (n = 3)*.

b *Not detected*.

## Conclusions

In this work, we used N-hydroxysuccinate active esterification to couple TC to carrier proteins and hence prepare artificial antigens, and finally obtain specific monoclonal antibodies. By using monoclonal antibodies to form signal probes with UCNP, a competitive immunochromatography procedure based on UCNPs was successfully developed for the simultaneous detection of various tetracycline antibiotic residues in milk. This procedure achieved an ultra-highly sensitive detection of TC, OTC, CTC and DOX within 10 min, with *IC*_50_ values of 0.32, 0.32, 0.26, 0.22 ng/mL, respectively. In addition, when testing spiked samples, their average recoveries were between 93.95 and 111.90%, with RSDs of < 9.95%. The detection of these analytes in actual samples using this procedure showed satisfactory results and had a high correlation with results obtained using HPLC. To the best of our knowledge, this is the first report on the detection of TC residues using UCNP-LFIC analysis. In short, the assay was shown to display high sensitivity, good stability, and good selectivity—and hence would be an effective method for screening antibiotics and other small molecules, and provide an alternative tool for food safety monitoring. At present, we are studying how to integrate more targets on the test strip to achieve multiple-target detection. We expect such multiple test strip analysis to find extensive applications in the field of food safety.

## Data Availability Statement

The raw data supporting the conclusions of this article will be made available by the authors, without undue reservation.

## Ethics Statement

The animal study was reviewed and approved by Animal Care and Use Committee of Hangzhou Normal University (Hangzhou, China).

## Author Contributions

MZ and CS designed the experiments. YX, EC, and BM conducted experiments and analyzed data. YX and EC wrote the main manuscript text and prepared all figures and tables. All authors reviewed the manuscript.

## Conflict of Interest

The authors declare that the research was conducted in the absence of any commercial or financial relationships that could be construed as a potential conflict of interest.

## Supplementary Material

Supplementary Figure 1 SDS-PAGE analysis of the artificial TC antigens. **(A)** SDS-PAGE analysis of TC-BSA. **(B)** SDS-PAGE analysis of TC-HSA.

Supplementary Figure 2 UV spectra of the artificial TC antigens. **(A)** UV spectrum of TC-HSA. **(B)** UV spectrum of TC-BSA.

Supplementary Table 1 Cross-reactivities of anti-TC mAb with antibiotics of the same family and other antibiotics.

Supplementary Table 2 Comparison of the analytical performances of optical biosensors for the detection of antibiotics.

Supplementary Table 3 Fluorescence spectrum data for the UNCPs before and after they having been labeled with antibody.

Supplementary Table 4 Test data of the fluorescence intensity of different batches of antibody-UNCP conjugate.
