# Supplementary figures and images for: Functional Up-Conversion Nanoparticle-Based Immunochromatography Assay for Simultaneous and Sensitive Detection of Residues of Four Tetracycline Antibiotics in Milk
Source: Front Chem. 2020 Oct 8;8:759. doi: 10.3389/fchem.2020.00759 (PMC7578426; doi:10.3389/fchem.2020.00759)

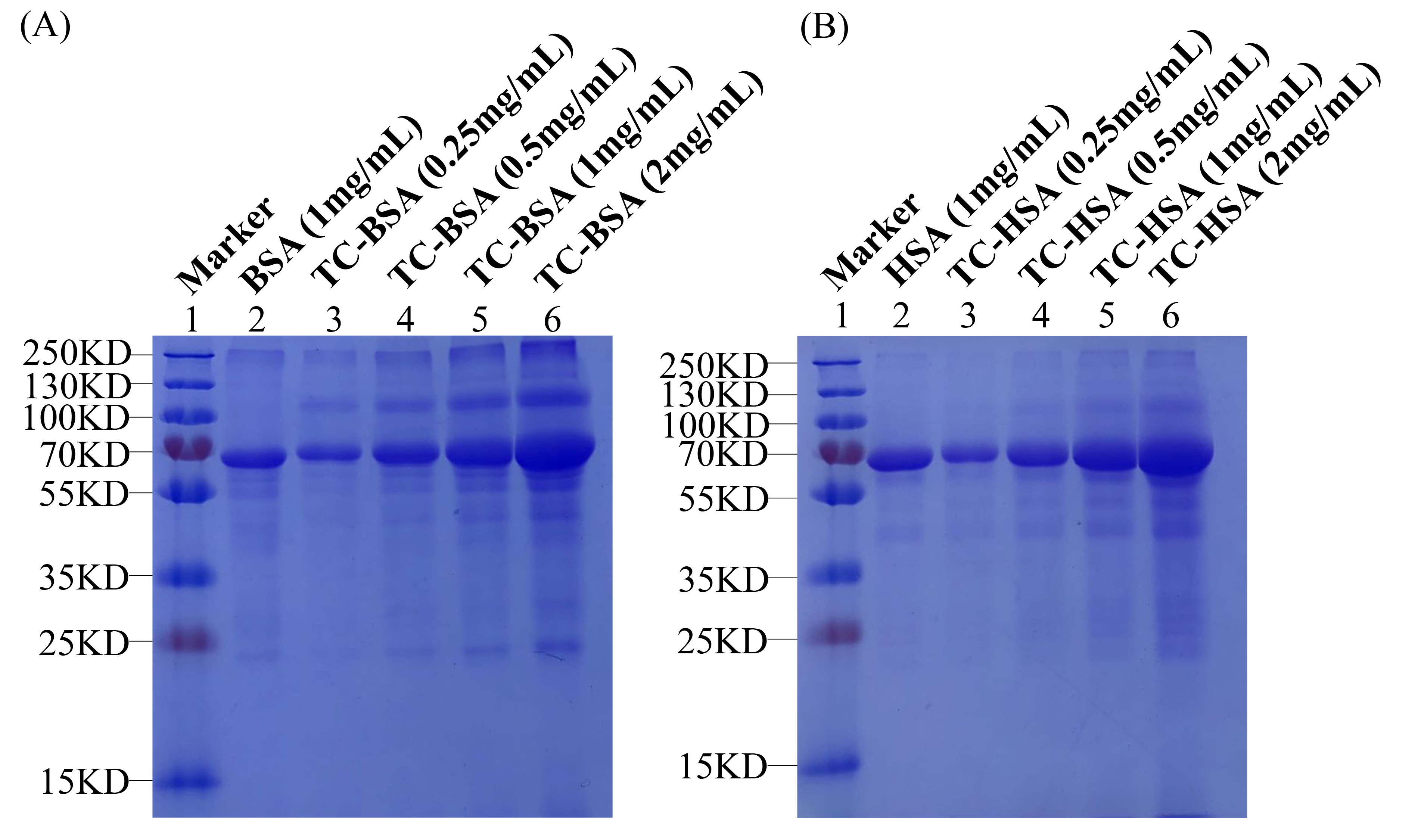

Supplement: Supplementary Figure 1 — SDS-PAGE analysis of the artificial TC antigens. (A) SDS-PAGE analysis of TC-BSA. (B) SDS-PAGE analysis of TC-HSA. [file Image_1.TIF]

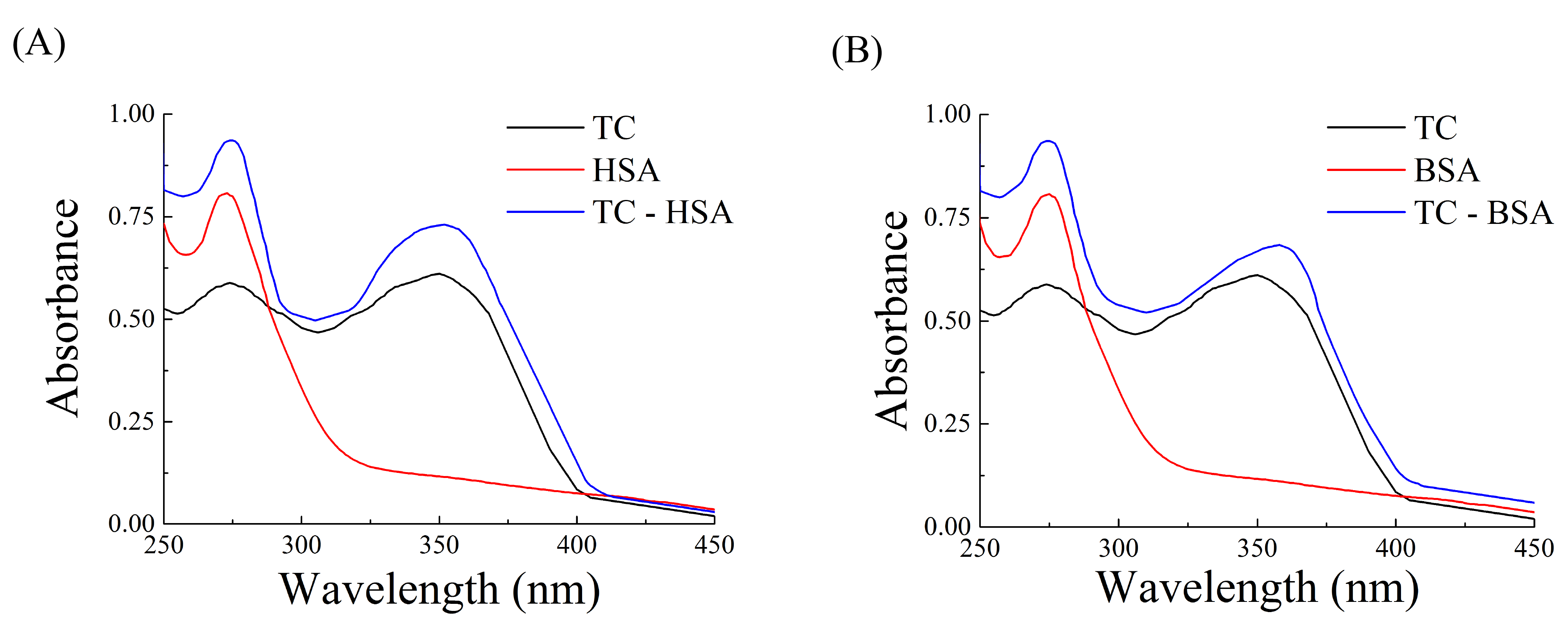

Supplement: Supplementary Figure 2 — UV spectra of the artificial TC antigens. (A) UV spectrum of TC-HSA. (B) UV spectrum of TC-BSA. [file Image_2.TIF]
