# Supplementary material for: Functional Up-Conversion Nanoparticle-Based Immunochromatography Assay for Simultaneous and Sensitive Detection of Residues of Four Tetracycline Antibiotics in Milk
Source: Front Chem. 2020 Oct 8;8:759. doi: 10.3389/fchem.2020.00759 (PMC7578426; doi:10.3389/fchem.2020.00759)
Supplement: Supplementary Table 1 — Cross-reactivities of anti-TC mAb with antibiotics of the same family and other antibiotics. [file Table_1.docx]

To determine the cross-reactivity of this anti-TC mAb, indirect competitive ELISA was carried out with antibiotics of the same family and other antibiotics including KAN, SM, ENR, PEN, FFC, TAP, GEN, ERY, SMZ and LIN. The result indicated that this anti-TC mAb has good specificity, with no cross reactivity to other antibiotics (Table S1).

**Table S1**. Cross-reactivities of anti-TC mAb with antibiotics of the same family and other antibiotics.

| antibiotics | Cross-Reactivity (%) |
| --- | --- |
| TC | 100 |
| CTC | 60 |
| OTC | 80 |
| DOX | 75 |
| KAN | <0.1 |
| SM | <0.1 |
| ENR | <0.1 |
| PEN | <0.1 |
| FFC | <0.1 |
| TAP | <0.1 |
| GEN | <0.1 |
| ERY | <0.1 |
| SMZ | <0.1 |
| LIN | <0.1 |
