# Supplementary material for: Functional Up-Conversion Nanoparticle-Based Immunochromatography Assay for Simultaneous and Sensitive Detection of Residues of Four Tetracycline Antibiotics in Milk
Source: Front Chem. 2020 Oct 8;8:759. doi: 10.3389/fchem.2020.00759 (PMC7578426; doi:10.3389/fchem.2020.00759)
Supplement: Supplementary Table 2 — Comparison of the analytical performances of optical biosensors for the detection of antibiotics. [file Table_2.docx]

**Table S2**. Comparison of the analytical performances of optical biosensors for the detection of antibiotics.

| Detection methods | Analytes | Detection  time (minutes) | LOD | Selectivity | References |
| --- | --- | --- | --- | --- | --- |
| GO-FSP | Oxytetracycline | - | 25 ng/mL | excellent | Tan et al., 2016 |
| AuNPs-aptasensor | Kanamycin | - | 321pM | high | Ramezani et al., 2016 |
| QD-IFA | Streptomycin  Tetracycline  Penicillin G | 90 | 5 pg/mL  5 pg/mL  5 pg/mL | well | Song et al., 2015 |
| MNPs-CB | Oxytetracycline  Tetracycline  Doxycycline | - | 26 nM  45 nM  48 nM | good | Wang et al., 2016 |
| aptamer-AuNP- colorimetric | Tetracycline | - | 45.8 nM | high | He et al., 2013 |
| UCNP-LFIC | Tetracycline  Oxytetracycline  Chlortetracycline  Doxycycline | 10 | 0.032 ng/mL  0.045 ng/mL  0.029 ng/mL  0.022ng/mL | good | This study |

GO-FSP: fluorescent sensing platform based on graphene oxide (GO) hydrogel; AuNPs-aptasensor：gold nanoparticles-aptasensor；

QD-IFA: quantum dot immunofluorescence assay;

MNPs-CB: magnetic nanoparticles colorimetric biosensor;

aptamer-AuNP- colorimetric: aptamer-gold nanoparticle-based colorimetric method;

UCNP-LFIC: up-conversion nanoparticles lateral-flow immunochromatographic.

The limit of detection (LOD) was calculated as the mean of the measured content of blank different samples (n=20) plus three standard deviations (mean + 3SD) (Food and Agricultural Immunology, 1998, 10(4), 307-315). We have calculated the LODs of tetracycline, oxytetracycline, chlortetracycline and doxycycline according to this calculation method were 0.032, 0.045, 0.029, 0.022 ng/mL, respectively.

**References**

Cerni, L., Biancotto, G., Tondolo, A., and Bogoni, P. (1998). Dexamethasone and clenbuterol detection by enzyme immunoassay in Bovine Liver Tissue: A new multiresidue extraction procedure. Food & Agricultural Immunology, 10, 307-315. doi: 10.1080/09540109809354994.

He, L., Luo, Y. F., Zhi, W. T., and Zhou, P. (2013). Colorimetric sensing of tetracyclines in milk based on the assembly of cationic conjugated polymer-aggregated gold nanoparticles. Food Analytical Methods, 6(6), 1704-1711. doi: 10.1007/s12161-013-9577-9.

Ramezani, M., Danesh, N. M., Lavaee, P., Abnous, K., & Taghdisif, S. M. (2016). A selective and sensitive fluorescent apta-sensor for detection of kanamycin based on catalytic recycling activity of exonuclease Ⅲ and gold nanoparticles. Sensors and Actuators, 222, 1-7. doi: 10.1016/j.snb.2015.08.024.

Song, E. Q., Yu, M. Q., Wang, Y. Y., Hu, W. H., Cheng, D., Swihart, M. T., et al. (2015). Multi-color quantum dot-based fluorescence immunoassay array for simultaneous visual detection of multiple antibiotic residues in milk. Biosensors & Bioelectronics, 72, 320-325. doi: 10.1016/j.bios.2015.05.018.

Tan, B., Zhao, H. M., Du, L., Gan, X. R., and Quan, X. (2016). A versatile fluorescent biosensor based on target-responsive graphene oxide hydrogel for antibiotic detection. Biosensors and Bioelectronics, 83, 267-273. doi: [org/10.1016/j.bios.2016.04.065](https://doi.org/10.1016/j.bios.2016.04.065).

Wang, Y. L., Sun, Y. J., Dai, H. C., Ni, P. J., Jiang, S., Lu, W. D., et al. (2016). A colorimetric biosensor using Fe3O4 nanoparticles for highly sensitive and selective detection of tetracyclines. Sensors and Actuators B Chemical, 236, 621-626. doi: 10.1016/j.snb.2016.06.029.
