# Supplementary material for: Functional Up-Conversion Nanoparticle-Based Immunochromatography Assay for Simultaneous and Sensitive Detection of Residues of Four Tetracycline Antibiotics in Milk
Source: Front Chem. 2020 Oct 8;8:759. doi: 10.3389/fchem.2020.00759 (PMC7578426; doi:10.3389/fchem.2020.00759)
Supplement: Supplementary Table 3 — Fluorescence spectrum data for the UNCPs before and after they having been labeled with antibody. [file Table_3.docx]

**Table S3**. Fluorescence spectrum data for the UNCPs before and after they having been labeled with antibody.

| Wavelengt (nm) | The fluorescence intensity | | Wavelengt  (nm) | The fluorescence intensity | |
| --- | --- | --- | --- | --- | --- |
|  | before | after |  | before | after |
| 350 | 6340 | 6037 | 476 | 951295 | 927933 |
| 351 | 7174 | 6825 | 477 | 911188 | 868659 |
| 352 | 6388 | 6004 | 478 | 855750 | 815047 |
| 353 | 6318 | 6001 | 479 | 789710 | 752804 |
| 354 | 6454 | 6042 | 480 | 721013 | 688522 |
| 355 | 7864 | 7480 | 481 | 646221 | 615926 |
| 356 | 7760 | 7278 | 482 | 569097 | 544382 |
| 357 | 7698 | 7281 | 483 | 496460 | 473039 |
| 358 | 6770 | 6486 | 484 | 431553 | 411321 |
| 359 | 6576 | 6289 | 485 | 365015 | 347508 |
| 360 | 6442 | 6098 | 486 | 307604 | 295198 |
| 361 | 7498 | 7111 | 487 | 256710 | 244708 |
| 362 | 7546 | 7152 | 488 | 211418 | 203438 |
| 363 | 7624 | 7370 | 489 | 176980 | 169707 |
| 364 | 7472 | 7006 | 490 | 144495 | 137733 |
| 365 | 7616 | 7216 | 491 | 117616 | 112429 |
| 366 | 7672 | 7252 | 492 | 96960 | 91905 |
| 367 | 7418 | 7062 | 493 | 78488 | 73646 |
| 368 | 7592 | 7294 | 494 | 65083 | 61985 |
| 369 | 7602 | 7284 | 495 | 53165 | 50524 |
| 370 | 11174 | 10713 | 496 | 43021 | 41273 |
| 371 | 11226 | 10748 | 497 | 35151 | 323310 |
| 372 | 12022 | 11411 | 498 | 29532 | 28022 |
| 373 | 12704 | 12090 | 499 | 24386 | 23190 |
| 374 | 13726 | 13175 | 500 | 20393 | 19498 |
| 375 | 15072 | 14347 | 501 | 17246 | 16452 |
| 376 | 17454 | 16504 | 502 | 15090 | 14250 |
| 377 | 20138 | 19007 | 503 | 12969 | 12387 |
| 378 | 20326 | 19215 | 504 | 11012 | 10547 |
| 379 | 22154 | 21020 | 505 | 10694 | 10136 |
| 380 | 25442 | 24900 | 506 | 9600 | 9228 |
| 381 | 26802 | 25560 | 507 | 9087 | 8546 |
| 382 | 33358 | 31642 | 508 | 8461 | 7945 |
| 383 | 38007 | 36550 | 509 | 8154 | 7770 |
| 384 | 44691 | 43530 | 510 | 7582 | 7280 |
| 385 | 53578 | 50364 | 511 | 7338 | 7039 |
| 386 | 58018 | 55386 | 512 | 7314 | 7032 |
| 387 | 64247 | 61185 | 513 | 7234 | 6810 |
| 388 | 70691 | 67435 | 514 | 7387 | 6959 |
| 389 | 78158 | 74404 | 515 | 7554 | 7139 |
| 390 | 86222 | 81909 | 516 | 7356 | 6933 |
| 391 | 91358 | 86464 | 517 | 7369 | 6904 |
| 392 | 86222 | 81918 | 518 | 7417 | 7084 |
| 393 | 78158 | 74404 | 519 | 7493 | 7295 |
| 394 | 70691 | 67435 | 520 | 7344 | 7090 |
| 395 | 64247 | 60785 | 521 | 7485 | 7266 |
| 396 | 58018 | 55386 | 522 | 7051 | 6885 |
| 397 | 53578 | 50364 | 523 | 6297 | 6052 |
| 398 | 44691 | 43530 | 524 | 6575 | 6243 |
| 399 | 38007 | 36550 | 525 | 6288 | 6021 |
| 400 | 33358 | 30086 | 526 | 6408 | 5973 |
| 401 | 26802 | 25560 | 527 | 7068 | 6737 |
| 402 | 25442 | 24803 | 528 | 6386 | 6085 |
| 403 | 22154 | 20483 | 529 | 6370 | 5917 |
| 404 | 20418 | 18403 | 530 | 6343 | 6097 |
| 405 | 17454 | 16439 | 531 | 6404 | 6111 |
| 406 | 15072 | 14436 | 532 | 6374 | 6075 |
| 407 | 13726 | 13049 | 533 | 6372 | 6062 |
| 408 | 11226 | 10676 | 534 | 6386 | 6095 |
| 409 | 11174 | 10798 | 535 | 6433 | 5953 |
| 410 | 10912 | 10224 | 536 | 6510 | 6243 |
| 411 | 10454 | 9864 | 537 | 6360 | 6021 |
| 412 | 9974 | 9523 | 538 | 6502 | 6170 |
| 413 | 9770 | 9380 | 539 | 6557 | 6170 |
| 414 | 9274 | 8865 | 540 | 6307 | 5947 |
| 415 | 9166 | 8794 | 541 | 6478 | 6234 |
| 416 | 8558 | 8073 | 542 | 6488 | 6223 |
| 417 | 8418 | 7822 | 543 | 6667 | 6328 |
| 418 | 8120 | 7727 | 544 | 6526 | 6124 |
| 419 | 7784 | 7283 | 545 | 6585 | 6273 |
| 420 | 7640 | 7219 | 546 | 6518 | 6210 |
| 421 | 7700 | 7364 | 547 | 6543 | 6504 |
| 422 | 7632 | 7337 | 548 | 6526 | 6514 |
| 423 | 7658 | 7295 | 549 | 6819 | 6783 |
| 424 | 7640 | 7258 | 550 | 6878 | 6599 |
| 425 | 7938 | 7432 | 551 | 7153 | 6810 |
| 426 | 7998 | 7415 | 552 | 7295 | 7021 |
| 427 | 8278 | 7807 | 553 | 7688 | 7429 |
| 428 | 8022 | 7604 | 554 | 7706 | 7443 |
| 429 | 8822 | 8307 | 555 | 6514 | 6272 |
| 430 | 8840 | 8332 | 556 | 7218 | 6877 |
| 431 | 7628 | 7338 | 557 | 6545 | 6299 |
| 432 | 8344 | 7991 | 558 | 8314 | 7920 |
| 433 | 7660 | 7232 | 559 | 10530 | 9919 |
| 434 | 9458 | 8802 | 560 | 14395 | 13648 |
| 435 | 8422 | 8058 | 561 | 19710 | 18859 |
| 436 | 8822 | 8384 | 562 | 26422 | 25399 |
| 437 | 6360 | 6168 | 563 | 33898 | 31726 |
| 438 | 6442 | 6198 | 564 | 41184 | 38066 |
| 439 | 6466 | 6040 | 565 | 47966 | 46215 |
| 440 | 6714 | 6383 | 566 | 52524 | 50376 |
| 441 | 7296 | 6800 | 567 | 53511 | 51025 |
| 442 | 7608 | 7240 | 568 | 52142 | 50339 |
| 443 | 8244 | 7866 | 569 | 46316 | 44161 |
| 444 | 8766 | 8358 | 570 | 39471 | 37960 |
| 445 | 9878 | 9278 | 571 | 33025 | 31389 |
| 446 | 11202 | 10694 | 572 | 25798 | 24631 |
| 447 | 13192 | 12559 | 573 | 21005 | 20062 |
| 448 | 15348 | 14446 | 574 | 16732 | 15890 |
| 449 | 17540 | 16739 | 575 | 13829 | 13104 |
| 450 | 20740 | 19765 | 576 | 11488 | 10600 |
| 451 | 24800 | 23307 | 577 | 9763 | 9295 |
| 452 | 30032 | 28004 | 578 | 8638 | 8263 |
| 453 | 35746 | 32766 | 579 | 7983 | 7571 |
| 454 | 43748 | 41833 | 580 | 7827 | 7485 |
| 455 | 54062 | 51208 | 581 | 7792 | 7457 |
| 456 | 66180 | 62824 | 582 | 7702 | 7369 |
| 457 | 79810 | 74641 | 583 | 7678 | 7131 |
| 458 | 98592 | 93146 | 584 | 7621 | 7101 |
| 459 | 119594 | 113946 | 585 | 7604 | 7118 |
| 460 | 146924 | 139590 | 586 | 7586 | 7098 |
| 461 | 179954 | 171994 | 587 | 7545 | 7071 |
| 462 | 214970 | 204533 | 588 | 7538 | 7052 |
| 463 | 261022 | 246933 | 589 | 7482 | 7000 |
| 464 | 312770 | 297600 | 590 | 7338 | 6954 |
| 465 | 371144 | 353600 | 591 | 7314 | 6967 |
| 466 | 438798 | 416400 | 592 | 7234 | 6810 |
| 467 | 504794 | 479214 | 593 | 7387 | 6959 |
| 468 | 578650 | 549067 | 594 | 7554 | 7139 |
| 469 | 657068 | 625733 | 595 | 7356 | 7131 |
| 470 | 733114 | 697867 | 596 | 7369 | 7120 |
| 471 | 802964 | 762933 | 597 | 7417 | 7084 |
| 472 | 870112 | 826000 | 598 | 7493 | 7142 |
| 473 | 926480 | 881463 | 599 | 7344 | 7019 |
| 474 | 967260 | 891069 | 600 | 7485 | 7124 |
| 475 | 998346 | 949064 |  |  |  |
