# Supplementary material for: Functional Up-Conversion Nanoparticle-Based Immunochromatography Assay for Simultaneous and Sensitive Detection of Residues of Four Tetracycline Antibiotics in Milk
Source: Front Chem. 2020 Oct 8;8:759. doi: 10.3389/fchem.2020.00759 (PMC7578426; doi:10.3389/fchem.2020.00759)
Supplement: Supplementary Table 4 — Test data of the fluorescence intensity of different batches of antibody-UNCP conjugate. [file Table_4.docx]

**Table S4**. Test data of the fluorescence intensity of different batches of antibody-UNCP conjugate.

| Batches | The fluorescence intensity | | Batches | The fluorescence intensity | |
| --- | --- | --- | --- | --- | --- |
|  | T line | C line |  | T line | C line |
| 1 | 3875  3681  3504  3478  3206  2998  2500  1532  1060  532  205  89  19 | 2677  2844  3131  3581  3775  3793  3909  3928  3994  4040  3975  3988  3976 | 2 | 3889  3693  3515  3489  3215  3005  2504  1530  1055  523  193  77  6 | 2682  2850  3139  3593  3788  3806  3923  3942  4008  4055  3989  4002  3990 |
